# Inkjet Printing of Heterostructures: Investigation and Strategies for Control of Interfaces

**DOI:** 10.1021/acsami.4c21170

**Published:** 2025-03-07

**Authors:** Jonathan S. Austin, Yundong Zhou, Geoffrey Rivers, Negar Gilani, Feiran Wang, Christopher J. Tuck, Ian S. Gilmore, Richard J. M. Hague, Gustavo F. Trindade, Lyudmila Turyanska

**Affiliations:** † Centre for Additive Manufacturing, Faculty of Engineering, University of Nottingham, Jubilee Campus, Nottingham NG8 1BB, U.K.; ‡ National Physical Laboratory, Teddington, Middlesex TW11 0LW, U.K.

**Keywords:** inkjet printing, heterostructures, multimaterial, graphene, perovskite CsPbBr_3_, PEDOT:PSS

## Abstract

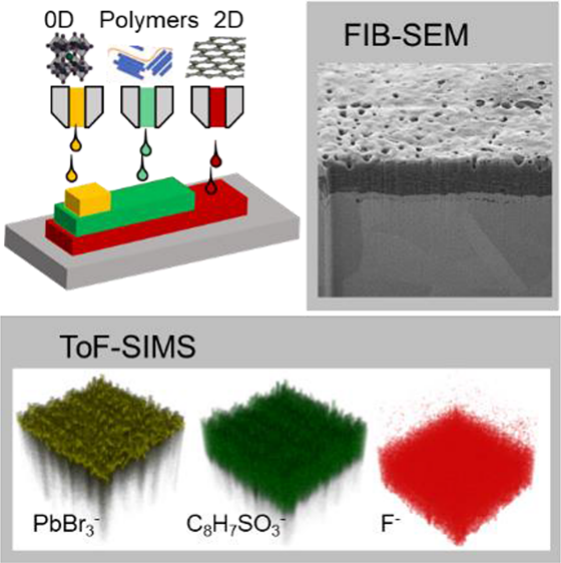

Development of printed electronics requires understanding and control of the interfaces in heterostructure devices. However, investigation of the interfaces between dissimilar materials to achieve control of intermixing presents challenges. Here, we report investigation of interfaces in inkjet printed heterostructures by time-of-flight secondary ion mass spectrometry (ToF-SIMS), focused ion beam scanning electron microscopy (FIB-SEM), and energy dispersive X-ray (EDX) analysis to provide complementary insights into the intermixing phenomena. By examining various heterostructures of 0D (CsPbBr_3_ nanocrystal), 2D (inkjet printed graphene, iGr), and polymeric (PEDOT:PSS) materials deposited with different printing parameters, we established the effect of ink composition and printing parameters on the intermixing depth. We demonstrated that in the heterostructures where the intermixing is dominated by layer porosity, the intermixing depth does not affect the electrical properties of the device, while intermixing by layer redispersion results in the decrease of the effective layer thickness accompanied by an increase of electrical resistance. The strategy for control over the interfacial composition and morphology in printed heterostructures could enable improved design and performance of printed devices.

## Introduction

Inkjet printed electronic devices have garnered significant interest in recent years, as inkjet deposition offers a scalable and customizable manufacturing route, with a wide range of available materials and substrates, for next-generation devices.^1,2^ The manufacturing of electronic devices often requires the interfacing of dissimilar materials into heterostructures with controlled geometries, and with inkjet printing, materials can be deposited into different architectures to enable a variety of distinct device functionalities, such as LEDs,^3,4^ solar cells,^5^ and chemical sensors.^6^ However, the fabrication of fully printed heterostructures with defined interfaces remains challenging due to intermixing of materials between layers.^7,8^ This is a common problem in solution processing techniques^9^ and occurs due to redispersion/dissolution of previously printed layers during deposition of subsequent layers.^10^ Moreover, surface roughness of inkjet-printed films caused by phenomena such as the coffee-ring effect, where the flow within the droplet leads to deposition of the suspended particles toward the edge in a ring-like pattern, can further affect the interfaces and hamper the performance of printed heterostructure devices.^11^ To address these challenges, several approaches were proposed: for example, orthogonal solvent systems^3,12,13^ or cross-linking agents^14,15^ were employed to limit redispersion of previous layers, and additional buffer layers were used to prevent intermixing.^16,17^

Despite a significant need to produce heterostructure devices to realize the potential of additive manufacturing of electronics,^2−5^ there is still limited understanding of the intermixing process during printing, largely due to the lack of accurate experimental methods to examine the interfaces. Time-of-flight secondary ion mass spectrometry (ToF-SIMS) is a powerful technique to chemically and spatially probe such heterostructures and investigate new methods to reduce intermixing, owing to its high spatial resolution, sensitivity, and molecular specificity.^18−21^ However, the roughness of inkjet printed surfaces may induce artifacts in ToF-SIMS data that may become indistinguishable from materials intermixing at interfaces. Therefore, other imaging techniques can be used alongside ToF-SIMS, such as focused ion beam scanning electron microscopy with energy dispersive X-ray analysis (FIB-SEM with EDX), which can remove ambiguities by giving additional information about the layer morphology, thickness, and elemental composition.

In this study, we employ ToF-SIMS, FIB-SEM, and EDX techniques in tandem to investigate the interfaces in inkjet printed heterostructures with 0D (CsPbBr_3_ perovskite nanocrystal), 2D (graphene, iGr), and polymeric (PEDOT:PSS) layers. The effect of ink formulations, printing strategies, and post-processing techniques on the intermixing in printed heterostructures is examined to achieve the control of interface quality. The primary mechanisms influencing intermixing were found to be a partial dissolution of the material layers during deposition and the intrinsic porosity of the printed layers. We demonstrate that substrate temperature and the number of printed layers of inkjet graphene affect the porosity of printed graphene films, which in turn influences the extent of intermixing through the graphene. Compositional analysis of ToF-SIMS data for iGr/PEDOT:PSS, iGr/CsPbBr_3_, and iGr/PEDOT:PSS/CsPbBr_3_ were corroborated with SEM and EDX studies and enabled us to investigate the interfaces in the inkjet printed structures. This work gives a blueprint for the characterization and optimization of interfaces in printed heterostructures and offers new insights into the manufacturing of printed electronic and optoelectronic devices.

## Experimental Methods

### Substrates

Prime-grade silicon wafers (200 nm SiO_2_ thickness, PI-KEM) and Al_2_O_3_ sapphire substrates (0.8 mm thickness, PI-KEM) were cleaned by sonicating in acetone for 30 min at room temperature followed by washing with IPA and drying with N_2_. Flexible polyimide (Kapton HN, DUPONT) and tempered Cu substrates (1 mm thickness, 99.9% purity, Advent Research Materials) were rinsed with IPA and dried under N_2_.

### Synthesis of CsPbBr_3_ NCs

A Cs-oleate precursor was made by adding 1.2 mmol of Cs_2_CO_3_ together with 18 mL of octadecene (ODE) and 2 mL of oleic acid (OA) to a three-necked flask and degassed at 120 °C for 30 min. Then the temperature was increased to 150 °C for 10 min, and a clear and transparent Cs-oleate solution was formed. CsPbBr_3_ NCs are synthesized using a modified hot-injection method.^33^ 1 mmol of PbO and 3 mmol of phenacyl bromide were mixed with 5 mL of OA and 25 mL of ODE in a 100 mL three-neck flask and degassed under nitrogen for 30 min at 120 °C. The temperature was then increased to 220 °C and 2.5 mL of OA was injected. The solution was annealed for about 20 min. After that, the temperature was lowered to 195 °C and 2.5 mL of Cs-oleate was injected. The solution was kept for 5 min at a temperature of 195 °C and cooled to room temperature by an ice water bath. To obtain the final product, the compound powder was purified by washing twice with isopropyl alcohol and dried under vacuum. The red CsPb(Br/I)_3_ QDs were prepared through an ion exchange method, with the PbI_2_–OA as an I-ion precursor.

### Ink Formulations

All materials, unless stated otherwise, were purchased from Sigma (UK) and used as received. iGr ink was printed as purchased (product number: 793663), consisting of liquid exfoliated graphene flakes (average size of 2590 nm^2^ and average thickness of 3 nm) encapsulated in ethyl cellulose (EC) dispersed into an 85:15 mixture of cyclohexanone/terpineol. PEDOT:PSS ink was formulated as previously reported in ref (22), by mixing PEDOT:PSS (Clevios, PH 1000, 31.8 wt %), deionized water (58.3 wt %), glycidoxypropyltrimethoxysilane (GOPS) (0.34 wt %), Cyrene (5.1 wt %), *n*-butanol (3.0 wt %), and polysorbate-80 (Tween-80) (0.67 or 0.30 wt %). The ink was mixed in air at room temperature for 20 h and filtered with a syringe filter (0.45 μm, Millipore Millex-LCR hydrophilic) before printing. CsPbBr_3_ perovskite NC inks were formulated as previously reported in ref (8) by dispersing 5 mg mL^–1^ CsPbBr_3_ NCs in a mixture of hexane, cyclohexanone, and terpineol (1:3:1 v/v) and sonicating for 30 min at room temperature and stored at room temperature under an N_2_ atmosphere. The CsPbBr_3_–PVP ink was formulated by adding 5 mg mL^–1^ of polyvinylpyrrolidone (PVP) with molecular weight *M*_W_ = 40,000 to the CsPbBr_3_ ink and repeating the sonication step.

### Inkjet Printing Process

All inks were deposited using a piezo-driven Fujifilm Dimatix DMP-2831 inkjet printer. iGr ink was printed by using 10 pL drop volume Dimatix DMC-11610 cartridges at 20 μm drop spacing. The printer was paused for 30 s between printing each layer to allow for drying, and nozzles were purged before printing each layer and periodically during printing (for 0.1 s every 100 printed swaths) to achieve consistent jetting. The substrate temperature during printing was investigated at *T*_subs_ = 20 °C and *T*_subs_ = 60 °C. After printing, films were annealed in air at *T*_ann_ = 250 °C for 2 h. PEDOT:PSS ink was printed using 2.4 pL drop volume Samba cartridges with a 20 μm drop spacing. The substrate temperature during printing was *T*_subs_ = 45 °C and nozzles were purged for 0.2 s before each layer. After printing, films were annealed in air at *T*_ann_ = 150 °C for 30 min. Perovskite inks were printed using 2.4 pL drop volume Samba cartridges under an N_2_ atmosphere with 20 μm drop spacing and *T*_subs_ = 60 °C. The nozzles were purged for 0.1 s every layer and films were dried on the print bed for 30 min after printing.^8^

### Time-of-Flight Secondary Ion Mass Spectrometry (ToF-SIMS)

**Figure 1:**
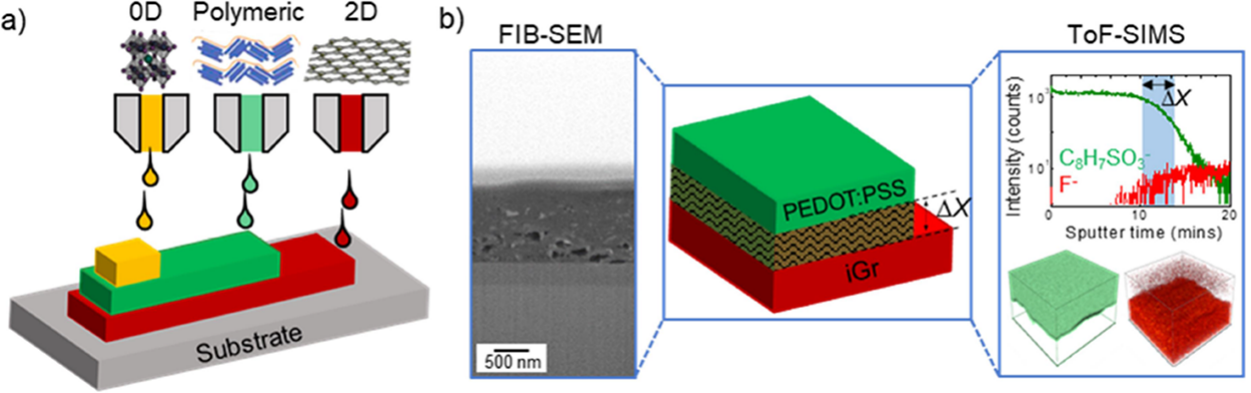
(a) Schematic diagram of inkjet deposition of a multimaterial 2D/polymer/0D heterostructure. (b) Diagram of a cross-section of a printed iGr/PEDOT:PSS heterostructure with the intermixing depth Δ*X* imaged by FIB-SEM (left inset) and by ToF-SIM (right inset) where the C_8_H_7_SO_3_^–^ signal (green) corresponds to PEDOT:PSS while the F^–^ signal (red) corresponds to iGr.

**Figure 2:**
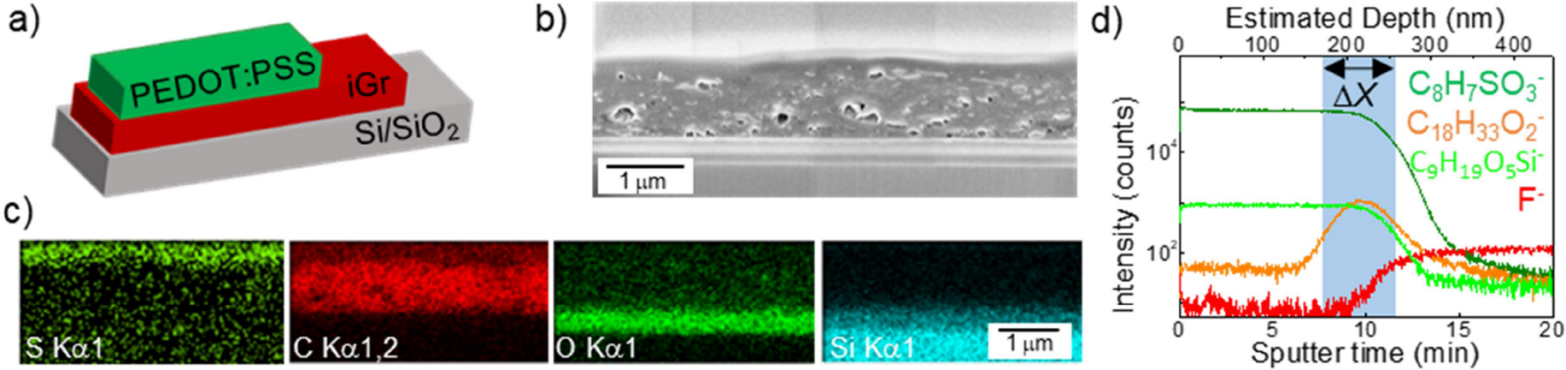
(a) Schematic diagram of the printed iGr/PEDOT:PSS heterostructure on the Si/SiO_2_ substrate, (b) cross-sectional SEM image, and (c) EDX mapping (iGr printed with *N*_L_ = 10, *T*_sub_ = 20 °C and PEDOT:PSS printed with *N*_L_ = 10, *T*_sub_ = 45 °C). (d) ToF-SIMS depth profile of the iGr/PEDOT:PSS printed on the Kapton substrate, where signals C_18_H_33_O_2_^–^ (Tween-80. 0.30 wt %), C_9_H_19_O_5_Si^–^ (GOPS), and C_8_H_7_SO_3_^–^ (PEDOT:PSS) correspond to components of the PEDOT:PSS ink, while F^–^ corresponds to iGr.

**Figure 3:**
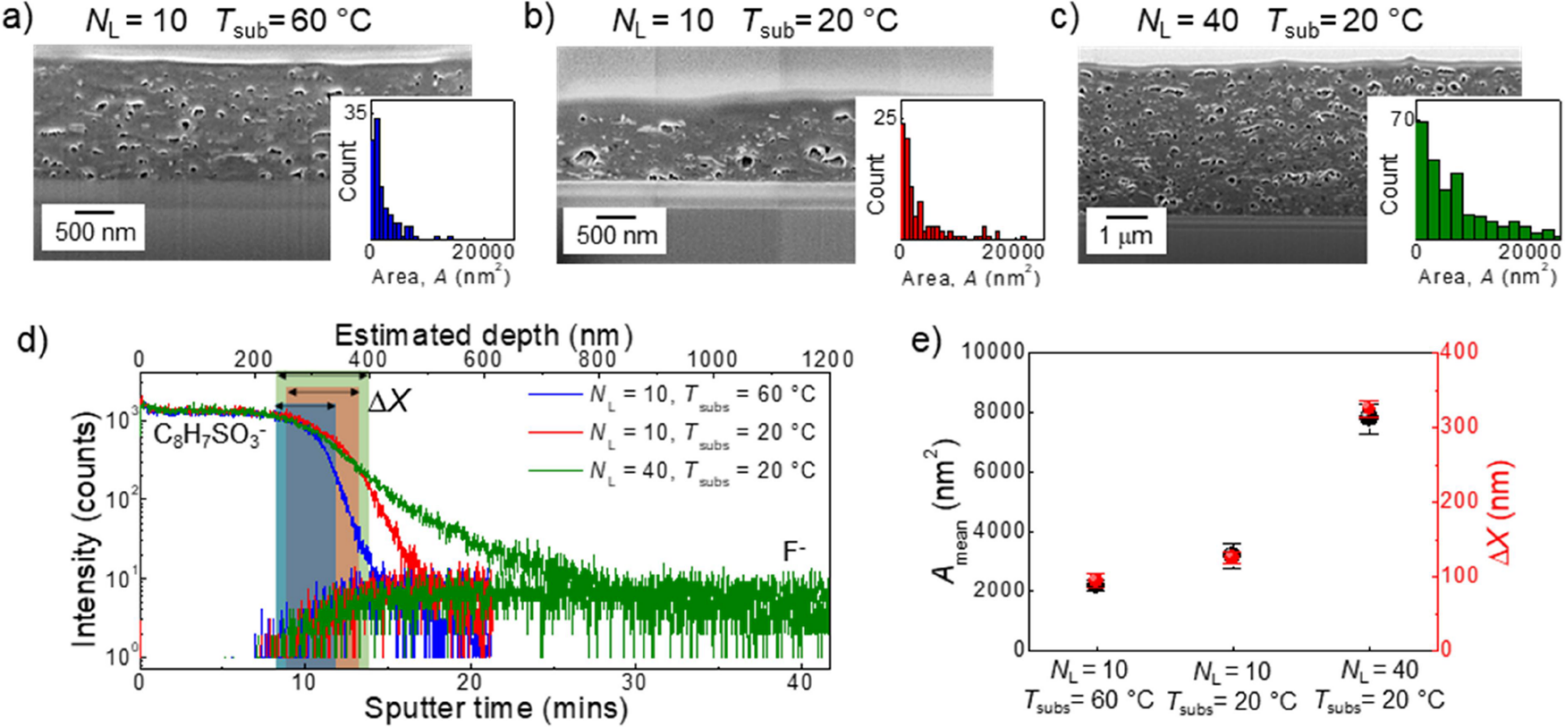
Representative cross-section SEM images of iGr/PEDOT:PSS (Si/SiO_2_ substrate, PEDOT:PSS *N*_L_ = 10) with iGr printed with the following parameters: (a) *N*_L_ = 10 and *T*_sub_ = 60 °C (b) *N*_L_ = 10 and *T*_sub_ = 20 °C, and (c) *N*_L_ = 40 and *T*_sub_ = 20 °C. Insets: Histograms of the cross-sectional area of the pores in the iGr layers. (d) Representative ToF-SIMS depth profiles of iGr/PEDOT:PSS samples in (a–c) represented by F^–^ signal for iGr and C_8_H_7_SO_3_^–^ signal for PEDOT:PSS. (e) Dependence of the average pore size and the intermixing depth Δ*X* on the printing parameters. Error bars for *A*_mean_ represent standard error of the mean and error bars for Δ*X* were calculated from at least three independent repeat measurements in different locations.

For data in Figures 2, 3 and 5, ToF-SIMS 3D chemical mapping was carried out using an IONTOF 5 instrument from IONTOF GmbH. The ToF-SIMS data were acquired in negative ion polarity mode by raster scanning a 30 keV Bi_3_^+^ primary ion beam and delivering 0.20 pA with a cycle time of 200 μs. A 10 keV GCIB gun was used as the sputter gun with an Ar_2000_^+^ beam delivering 1.05 nA. The raster size was 400 × 400 μm^2^. A low-energy (20 eV) electron flood gun was employed to neutralize charge build up. The field of view was 200 × 200 μm^2^ for Figures 1 and 2, 31 × 31 μm^2^ for Figure 3, and, 30 × 30 μm^2^ for Figure 5. In all measurements, a constant etching speed was used for the duration of depth profiling.

**Figure 4:**
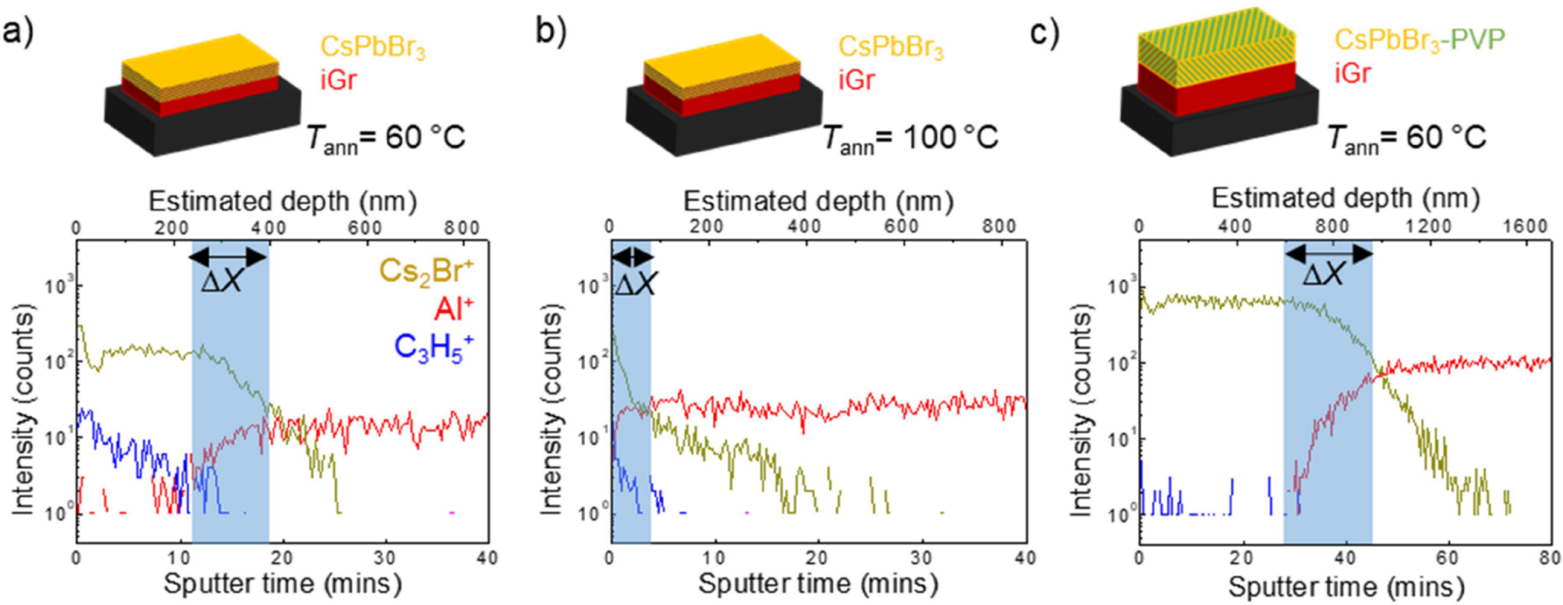
Schematic diagrams and ToF-SIMS positive polarity mode depth profiles of iGr/CsPbBr_3_ represented by Al^+^ and Cs_2_Br^+^ signals, respectively (*N*_L_ = 10 layers for iGr and for CsPbBr_3_, sapphire substrate). Signal C_3_H_5_^+^ represents residual organic solvents in the perovskite layer. Representative results are shown for perovskite layer annealed at (a) *T*_ann_ = 60 °C for 30 min and (b) *T*_ann_ = 100 °C for 30 min, and with (c) CsPbBr_3_–PVP layer annealed at *T*_ann_ = 60 °C for 30 min. All measurements were repeated on at least three different areas of each sample.

For data in Figure 4, ToF-SIMS 3D chemical mapping of the PNCs/iGr samples was carried out using an OrbiSIMS instrument from IONTOF GmbH.^34^ The ToF-SIMS data were acquired in positive ion polarity mode by raster scanning a 30 keV Bi_3_^+^ primary ion beam and delivering a 0.18 pA peak with a cycle time of 200 μs. For Figure 4a,b, a 2 keV O_2_^+^ beam with a current of 20 nA was used for sputtering in the interlaced mode. For Figure c, noninterlaced mode was used, also with a 2 keV O_2_^+^ beam with a current of 20 nA. The raster size was 300 × 300 μm^2^. A low-energy (20 eV) electron flood gun was employed to neutralize charge buildup. The field of view was 12 × 12 μm^2^ for Figure 4a–c.

The depth in profiles was estimated using SEM measurements of PEDOT:PSS or iGr layer thickness and extrapolating the calculated sputter rate over the whole profile. For top heterostructure layers, thickness in depth profiles was determined by the sputter time taken for a signal to drop to 16% of its maximum intensity. For lower heterostructure layers, this was the time between the points at which the signal first and last reached 16% of its maximum intensity. Intermixing length, Δ*X*, between two materials was defined as the distance between 84 and 16% of the maximum signal intensity of the top layer of the heterostructure.^24^ Error was calculated from standard deviation of three repeat measurements of Δ*X* in different locations.

### FIB SEM and EDX

A ZEISS Crossbeam 550 FIB-SEM (Oberkochen, Germany) instrument was used to prepare cross sections of the samples and capture SEM images. In some samples, to protect the top layer from Ga^+^ implantation, platinum pads (15 μm × 3 μm) were deposited using the gas injection system (GIS) within the FIB prior to cross-sectioning. Subsequently, the FIB, operating at a current of 300 pA, was employed to section the samples. SEM images were acquired using the secondary electron secondary ion (SESI) and Inlens SE detectors, operated at 2 kV and 200 pA, with a working distance of 5 mm. The elemental composition of the interfaces was analyzed using an Energy-Dispersive X-ray (EDX) spectrometer (Oxford Instruments) integrated within the FIB-SEM chamber, at a working distance of 5 mm and a beam voltage of 5 kV. Analysis of pore sizes was performed using ImageJ.^35^

### Statistical Analysis

All data reported in this paper are shown as an average value and standard deviation of at least three independent measurements.

## Results and Discussion

To explore printed heterostructure interfaces, a number of heterostructures were produced by drop-on-demand (DoD) inkjet deposition of in-house formulated all-inorganic CsPbBr_3_ perovskite nanocrystal (NC)^8^ and PEDOT:PSS^22^ inks, as well as commercially available graphene ink (iGr) (Figure 1a). Printed heterostructures were probed by a combination of FIB-SEM and ToF-SIMS techniques (Figure 1b), providing complementary insights into thickness, porosity, and internal structure of the layers and interfaces with high-resolution compositional analysis.

In inkjet printed heterostructures, the interfaces are defined by the morphology of the printed layers, which is influenced by the dissolution and diffusion of the consecutively deposited inks. This process results in intermixing of the materials in successive layers. The degree of intermixing depends on the composition and the morphology of printed layers, which can be modified and controlled by the deposition and postdeposition strategies. To disentangle these effects, we first investigated iGr/PEDOT:PSS heterostructures (Figure 2a). The water-based PEDOT:PSS ink contained the co-solvents cyrene and glycerol carbonate (GC),^22^ whereas the iGr ink contained the nonpolar solvents cyclohexanone and terpineol. To fabricate the heterostructure, *N*_L_ = 10 printed layers of iGr were deposited at a substrate temperature *T*_sub_ = 20 °C and then annealed at *T*_ann_ = 250 °C in air for *t* = 2 h. Subsequently, *N*_L_ = 10 printed layers of PEDOT:PSS were printed onto the iGr at *T*_sub_ = 45 °C and annealed at *T*_ann_ = 150 °C in air for *t* = 30 min. The iGr layer had a thickness of 1020 ± 40 nm with some porosity, while PEDOT:PSS formed a continuous layer with a thickness of 260 ± 40 nm, as estimated from the FIB-SEM imaging (Figure 2b). The elemental composition of the layers (Figure 2c) was not significantly distinct to observe and quantify intermixing. This is due to the lower sensitivity of SEM and EDX compared to ToF-SIMS as well as the high chemical similarity between graphene and PEDOT:PSS.

ToF-SIMS depth profiling offers high chemical specificity to distinguish the layers. The C_8_H_7_SO_3_^–^ signal is used to map the PEDOT:PSS and the F^–^ signal is used to map the graphene (SI1, Figure S1). Inorganic impurities (likely introduced to graphene during the exfoliation process), such as F, Al_,_ and Na, are commonly used marker signals in SIMS for carbon-based materials due to their ease of ionization.^23^ ToF-SIMS maps revealed a region of overlap between the PEDOT:PSS and iGr signals (Figure 2d), thus indicating a region of intermixing between the two layers. We estimate the intermixing depth, Δ*X*, using the International Union of Pure and Applied Chemistry (IUPAC) definition, as the distance between the 84 and 16% intensity.^24^ These limits correspond to the 2σ-value of the Gaussian distribution of the signal at the interface. However, the resolution combined with artifacts such as roughness (natural or beam-induced), lateral heterogeneity, and molecular/atomic intermixing in the *z*-direction also influence the estimate. In our sample, the intermixing depth Δ*X* = 90 ± 20 nm was estimated as a region of overlap between the C_8_H_7_SO_3_^–^ and F^–^ signals, corresponding to PEDOT:PSS and iGr, respectively (Figure 2d), over which the PEDOT:PSS signal decreased from 84 to 16% of its original value. We note that ToF-SIMS data analysis was performed within different fields of view (FOV), with areas of 200 × 200 μm^2^ and 32 × 32 μm^2^ (SI1, Figure S2). The size of the selected FOV covers the sample area with different surface roughness because the droplet size is ∼40 μm in diameter. Despite the differing surface roughness, the estimated signal overlap (i.e., intermixing depth) is comparable, confirming that the estimated value of Δ*X* we is independent of surface roughness (SI1, Figure S2), and confirming that the overlapping signals are caused by intermixing, rather than by the macroscopic surface morphology or lateral heterogeneity at the interface caused by differential ion beam sputtering and the inkjet-deposition coffee ring effect.

ToF-SIMS depth profiles also showed the presence of PEDOT:PSS ink additives. 3-glycidyloxypropyl-trimethoxysilane GOPS (signal C_9_H_19_O_5_Si^–^) was found to be evenly distributed throughout the printed PEDOT:PSS layer (Figure 2d), while the surfactant polysorbate-80 (Tween-80, signal C_18_H_33_O_2_^–^) was accumulated at the iGr/PEDOT:PSS interface. To explore the effect of this accumulation layer on intermixing, further samples were printed with the polysorbate-80 concentration increased from 0.30 to 0.67 wt % (SI1, Figure S3). This resulted in a significant increase in the intermixing depth to Δ*X* = 170 ± 40 nm.

It can be expected that the presence of pores and macroscopic defects can have a significant effect on the intermixing. Porosity within iGr was revealed in cross-section FIB-SEM studies and was found to be affected by the printing parameters, particularly by the substrate temperature and the number of printed layers (Figure 3a–c, SI1). For *N*_L_ = 10 printed layers, an increase in substrate temperature from *T*_sub_ = 20 °C (Figure 3b) to *T*_sub_ = 60 °C (Figure 3a) resulted in a decrease of the average pore area, *A*_mean_, and narrowing of the size distribution from *A*_mean_ = 3700 ± 400 nm^2^ to 2300 ± 200 nm^2^. We note that printing at higher *T*_sub_ = 60 °C results in greater ink pinning on the substrate and smaller drop spreading, hence a larger iGr layer thickness of 1470 ± 50 nm (Figure 3a). With an increasing number of layers to *N*_L_ = 40 (layer thickness 3540 ± 70 nm), the samples printed at *T*_sub_ = 20 °C have over 2-fold larger average pore size *A*_mean_ = 7800 ± 600 nm^2^ (Figure 3c). We envisage that higher substrate temperatures used during printing increase the rate of solvent evaporation, hence reducing the amount of entrapped solvent within the iGr, and the porosity is formed due to the removal of trapped solvents and additives during postdeposition annealing. In the samples produced with larger *N*_L_, the thickness of the layer is larger, which may impede solvent and additive removal, leading to the formation of larger pores. We note that while the porosity can be controlled by the printing strategy (*N*_L_, *T*_sub_, etc.), it does not significantly affect the electrical properties of the iGr printed with *N*_L_ > 10, where bulk-like conductivity dominates the charge transport^25^ (SI1, Figure S4).

ToF-SIMS depth profiling demonstrated the presence of PEDOT:PSS within the top part of the iGr layer (Figure 3d), with the depth of intermixing being affected by the printing parameters. For the iGr/PEDOT:PSS heterostructures produced with *N*_L_ = 10 printed iGr layers, an increase of *T*_sub_ from 20 to 60 °C led to a small decrease of the intermixing length by ∼20%, from Δ*X* = 126 ± 10 nm to Δ*X* = 95 ± 20 nm. An increase in the number of printed iGr layers from *N*_L_ = 10, (Figure 3d, red lines) to *N*_L_ = 40 (Figure 3d, green lines) led to a significant increase of the intermixing length to Δ*X* = 325 ± 20 nm. The relative change of Δ*X* for the three samples measured is well correlated with the average pore sizes in these samples (Figure 3e), leading us to conclude that intermixing depth estimated for these samples is dominated by the infilling of PEDOT:PSS into the iGr layer through the pores rather than partial dissolution of the iGr layer. The sharpness of the interfaces in the SEM micrographs (Figure 3a–c) is not directly comparable to the chemical depth profiles because (i) ToF-SIMS has higher sensitivity and resolution compared to SEM so is more likely to detect compositional changes within the intermixing region and (ii) in absolute terms, the presence of pores will cause a blurring artifact in the ToF-SIMS profiles, which makes them comparable across samples as long as the artifact is a constant (indeed, we note that these results were independent of the FOV used to analyze the ToF-SIMS data). Moreover, we note that intermixing of PEDOT:PSS into the iGr film had no significant effect on the sheet resistance of iGr, further confirming that infilling of the pores is the dominating effect, and the effective thickness of the iGr layer that defines its electrical properties was not significantly affected. We note that the sheet resistance of iGr is strongly affected by the flakes' packing density within the random network of individual flakes,^25^ and for iGr samples with 10 printed layers, the *R*_s_ = 500 ± 300 Ω/sq. The sheet resistance of PEDOT:PSS (*N*_L_ = 10) is *R*_s_ = 400 ± 200 Ω/sq (SI1, Figure S5).

Because the properties and composition of the inks affect the interface, we also examined the effect of deposition of perovskite CsPbBr_3_ NCs (average size 11 ± 1 nm)^8^ onto iGr, as the formulation of their liquid carriers is very similar (Figure 4a–c). The iGr was printed at *T*_sub_ = 20 °C and annealed at *T*_ann_ = 250 °C (30 min) in air before printing of the perovskite layer (*T*_sub_ = 60 °C, *T*_ann_ = 60 °C). ToF-SIMS was performed in positive polarity mode to map iGr/CsPbBr_3_ heterostructures with a Cs_2_Br^+^ signal used to map the CsPbBr_3_, Al^+^ signal to map the iGr, and C_3_H_5_^+^ signal to map residual organic solvents (SI2, Figure S6). Note that these samples were printed on sapphire; however, the Al^+^ signal was only used to map graphene above the substrate, which was observed using sapphire-specific ions such as Al_2_O^+^. The ToF-SIMS depth profiles (Figure 4a) revealed the intermixing depth of Δ*X* = 90 ± 50 nm. An increase of the annealing temperature of CsPbBr_3_ to *T*_ann_ = 100 °C (Figure 4b) led to a small decrease of the intermixing length to Δ*X* = 60 ± 10 nm, and a small reduction of the C_3_H_5_^+^ signal, corresponding to removal of entrapped organic solvents. These results indicate that the deposition of perovskite ink formulated with the same solvents as those used in iGr ink (cyclohexanone and terpineol) can lead to redispersion of iGr facilitating intermixing. Also, interdiffusion through the porous iGr layer is likely to occur due to small sizes of perovskite NCs (∼11 nm), as was previously demonstrated for other 0D materials such as AgNPs^26^ and FA_0.8_MA_0.2_Pb(I_0.8_Br_0.2_)_3_ NCs.^27^ We attribute the lack of a stabilization region, particularly for the sample annealed at *T*_ann_ = 100 °C, to greater intermixing with the iGr, resulting in thin and poorly defined perovskite layer. Intermixing between the CsPbBr_3_ NCs and iGr has significant impact on its electrical properties,^8^ with iGr sheet resistance increasing by >50% following overprinting with CsPbBr_3_ NCs (SI2, Figure S7). The increase in resistance is expected, as CsPbBr_3_ NCs films are insulating; hence, the intermixing leads to lower packing density of conductive graphene networks and reduces effective thickness of the iGr layer.

**Figure 5:**
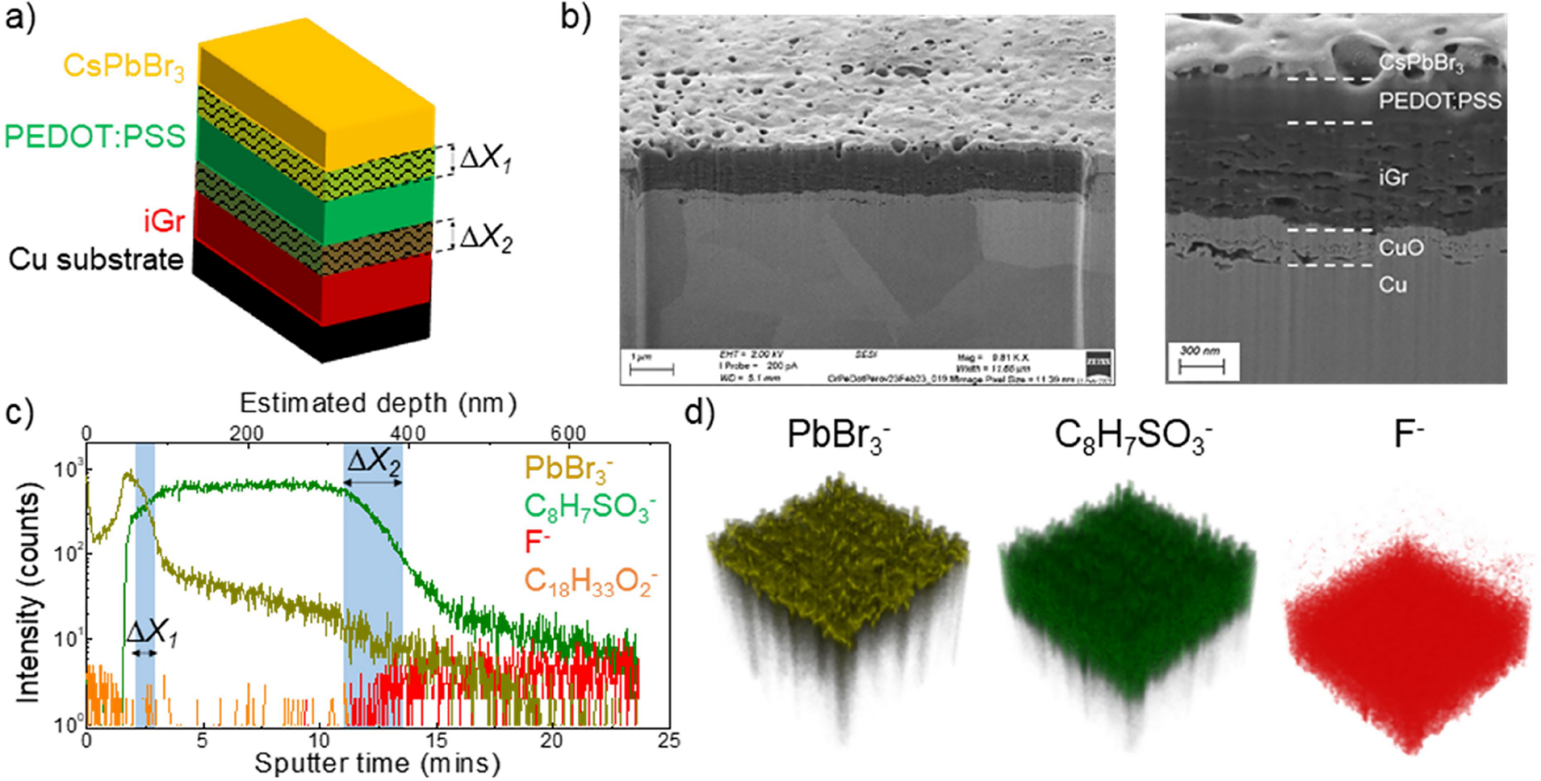
(a) Diagram of iGr/PEDOT:PSS/CsPbBr_3_ heterostructures showing intermixing between layers and (b) FIB-SEM cross-sectional images of the heterostructure on a Cu substrate (iGr: *N*_L_ = 10 layers, *T*_sub_ = 60 °C, and *T*_ann_ = 250 °C for *t* = 2 h, PEDOT:PSS: *N*_L_ = 10 layers, *T*_sub_ = 45 °C, and *T*_ann_ = 150 °C for *t* = 30 min, and CsPbBr_3_: *N*_L_ = 100 layers, *T*_sub_ = 60 °C, and *T*_ann_ = 60 °C for *t* = 30 min). (c) Representative ToF-SIMS depth profiles of iGr (F^–^), PEDOT:PSS (C_8_H_7_SO_3_^–^), and CsPbBr_3_ (PbBr_3_^–^) with 50 layers of CsPbBr_3_ on a Si/SiO_2_ substrate. (C_18_H_33_O_2_^–^) signal represents the Tween-80 surfactant in PEDOT:PSS ink. ToF-SIMS was performed in negative polarity mode with regions of intermixing denoted. (d) 3D ToF-SIMS reconstruction of the heterostructure over a 200 × 200 μm area in *X*–*Y* (the *Z*-axis is not to scale). All measurements were repeated on at least three different areas of each sample.

In order to control the layer formation and the depth of intermixing, the composition of inks was optimized. The addition of 5 mg/mL of the polymer poly vinylpyrrolidone (PVP) into the CsPbBr_3_ ink (CsPbBr_3_–PVP) (Figure 4c) greatly impacted the iGr/CsPbBr_3_ interface, with clear evidence of the CsPbBr_3_ layer and graphene signal detectable only at a depth of ∼600 nm, with an estimated intermixing depth of Δ*X* = 290 ± 100 nm. We propose that PVP can interact with hydrophobic NC capping ligands (e.g., oleic acid), either through ligand substitution or formation of a secondary ligand layer, which in turn alters the interaction of the CsPbBr_3_ NCs with the annealed iGr layer. Separation of PVP toward the top of inkjet printed layers after printing and annealing of the CsPbBr_3_ NCs agrees with previously reported behavior in AgNP-PVP inks.^28^ Deposition of CsPbBr_3_–PVP onto the iGr does not affect the electrical properties of the iGr films (SI2, Figure S8). Informed by the results on two-material structures, we produced three-material iGr/PEDOT:PSS/CsPbBr_3_ heterostructures (Figure 5a). FIB-SEM images (Figure 5b) and EDX maps (SI3, Figure S9) revealed the formation of three distinct layers, with some intermixing between the CsPbBr_3_ and PEDOT:PSS layers. For the samples with *N*_L_ = 100 layers of CsPbBr_3_ printed on top of *N*_L_ = 10 layers of PEDOT:PSS and iGr, we measured layer thicknesses of 180 ± 70 nm, 270 ± 40, and 710 ± 90 nm, respectively, via SEM. The iGr layer was printed at *T*_sub_ = 60 °C and had an average pore size of *A*_mean_ = 2000 ± 300 nm^2^, comparable to those in iGr/PEDOT:PSS (SI3, Figure S10). We note that the contact angle of iGr on Cu substrate (conductive substrate used for SEM studies) is smaller (θ = 21°) than on Si/SiO_2_ substrate (θ = 26°) (SI3, Figure S11), resulting in greater ink spreading and a larger print area. Hence, we observe thinner layers for the same *N*_L_ on Cu compared to those on Si/SiO_2_.

ToF-SIMS was performed in negative polarity mode to map the iGr/PEDOT:PSS/CsPbBr_3_ heterostructures (SI3, Figure S12) and revealed the CsPbBr_3_ signal over the depth of ∼80 nm above the PEDOT:PSS (Figure 5c). We envisage that the peak in the PbBr_3_^–^ profile just above the polymeric layer is due to PEDOT:PSS surface roughness, which is typically ∼50 nm for *N*_L_ = 4 layer PEDOT:PSS.^22^ The estimated intermixing depth at the PEDOT:PSS/CsPbBr_3_ interface was Δ*X*_1_ = 25 ± 10 nm, which may be an underestimate due to the matrix effect on the PbBr_3_^–^ signal.^29^ The iGr/PEDOT:PSS interface was well-defined with Δ*X*_2_ = 70 ± 10 nm. Interestingly, the polysorbate-80 surfactant signal, C_18_H_33_O_2_^–^ (Figure 5c) was observed at the top of the PEDOT:PSS layer at the PEDOT:PSS/CsPbBr_3_ interface, rather than at the iGr/PEDOT:PSS interface as was observed in iGr/PEDOT:PSS heterostructures (Figure 2e and SI1, Figure S3). This is likely due to the chemical composition of the perovskite layer, specifically the presence of oleic acid ligands, which haver the same mass^36^ of *m*/*z* = 281 as polysorbate-80, when measured with ToF-SIMS, and merits further studies. We also observed no significant change in the electrical properties of iGr following the deposition of CsPbBr_3_ NCs in the iGr/PEDOT:PSS/CsPbBr_3_ heterostructure (SI3, Figure S13). This agrees with the ToF-SIMS results and demonstrates that PEDOT:PSS can act as an effective barrier to control intermixing between the perovskite NCs and iGr.

To explain the observed results, we propose that the two key factors affecting intermixing are pore filling and redispersion. In iGr/PEDOT:PSS heterostructures, the two inks are based on different solvents, so redispersion of iGr is not significant. Thus, pore filling dominates the intermixing, as is evident from the correlation between the pore sizes and the intermixing depth. Because the packing of the graphene flakes is not changing, there is no effect of the intermixing on the electrical properties of iGr. In iGr/CsPbBr_3_ heterostructures, redispersion of the iGr occurs as the same solvents are used in both inks, leading to a decrease of the thickness of iGr layer with high flake packing density and hence increasing sheet resistance.^8,25^ Addition of the PVP to the CsPbBr_3_ ink PVP limits redispersion, and the pore filling (*A*_mean_ > 2000 nm^2^) dominates the intermixing; hence, there is no significant effect on electrical properties of iGr.

Our work focused on low-dimensional and polymeric materials, and the results demonstrate that the investigations of the intermixing by complementary techniques, ToF SIMS and SEM, provide important insights into the phenomena affecting the layer interfaces. We successfully used this for informed optimization of the ink composition and deposition parameters to control the properties of printed heterostructures. Detailed information on chemical composition achieved by ToF-SIMS was used before to probe devices such as heterostructure solar cells^21,30^ and OLEDs.^31,32^ However, we demonstrated that morphology of the layers has a significant impact on the intermixing, hence complementary information from mass spectrometry and electron microscopy is essential to understand the performance of printed heterostructures. We note that alternative methods, such as TEM and XPS can potentially be employed to probe the interfaces,^22,37^ with XPS potentially offering high specificity for inorganic heterostructures. In our work, we demonstrated that cross-sectional SEM and EDX with ToF-SIMS analysis provide an optimal combination of large field of view and molecular and elemental composition to investigate heterostructures composed of both organic and inorganic layers.

## Conclusions

Analysis of interfaces in additively manufactured, particularly inkjet printed, heterostructures is challenging. By combining complementary insights from SEM and ToF-SIMS analysis, we demonstrated the effect of the material formulations and printing strategies on the intermixing length at the interfaces. The developed approach can be successfully applied for analysis of dissimilar materials, as we exemplify for 2D/0D iGr/CsPbBr_3_, 2D/polymer iGr/PEDOT:PSS and polymer/0D PEDOT:PSS/CsPbBr_3_ interfaces. The approach developed here is transferable to any solution-processed heterostructure devices. This work is relevant for application in the fast-growing area of printed electronics as well as flexible and wearable electronics and can underpin the work of developing strategies for scalable printing of electronic and optoelectronic devices.

## Data Availability

All data are available from corresponding authors upon reasonable request.

## Supporting Information Available

SI1. Additional experimental results on ToF SIMS, FIB-SEM and electrical characterization studies of iGr/PEDOT:PSS heterostructures; SI2. Additional experimental results of ToF SIMS and electrical characterization studies of iGr/CsPbr_3_ heterostructures; SI3 Additional experimental results of iGr/PEDOT:PSS/CsPbr_3_ heterostructures, including EDX maps, pore size evaluation, contact angle measurements, ToF SIMS results, and electrical characterization (PDF)

## References

[ref1] Gao M.; Li L.; Song Y. Inkjet printing wearable electronic devices. J. Mater. Chem. C 2017, 5 (12), 2971–2993. 10.1039/C7TC00038C.

[ref2] Bastola A.; He Y.; Im J.; Rivers G.; Wang F.; Worsley R.; Austin J. S.; Nelson-Dummett O.; Wildman R. D.; Hague R.; Tuck C. J.; Turyanska L. Formulation of functional materials for inkjet printing: a pathway towards fully 3D printed electronics. Mater. Today Electron. 2023, 6, 100058 10.1016/j.mtelec.2023.100058.

[ref3] Zhao J.; Lo L.; Wan H.; Mao P.; Yu Z.; Wang C. High-speed fabrication of all-inkjet-printed organometallic halide perovskite light-emitting diodes on elastic substrates. Adv. Mater. 2021, 33 (48), 2102095 10.1002/adma.202102095. 34623708

[ref4] Vescio G.; Mathiazhagan G.; González-Torres S.; Sanchez-Diaz J.; Villaueva-Antolí A.; Sánchez R. S.; Gualdrón Reyes A. F.; Oszajca M.; Linardi F.; Hauser A.; Vinocour-Pacheco F. A.; Żuraw W.; Öz S.; Hernández S.; Mora-Seró I.; Cirera A.; Garrido B. Fully inkjet-printed green-emitting PEDOT:PSS/NiO/colloidal CsPbBr_3_ /SnO_2_ perovskite light-emitting diode on rigid and flexible substrates. Adv. Eng. Mater. 2023, 25 (21), 2300927 10.1002/adem.202300927.

[ref5] Verma A.; Martineau D.; Abdolhosseinzadeh S.; Heier J.; Nüesch F. Inkjet printed mesoscopic perovskite solar cells with custom design capability. Mater. Adv. 2020, 1 (2), 153–160. 10.1039/D0MA00077A.

[ref6] Van Tam T.; Hur S. H.; Chung J. S.; Choi W. M. Novel paper- and fiber optic-based fluorescent sensor for glucose detection using aniline-functionalized graphene quantum dots. Sens. Actuators, B 2021, 329, 129250 10.1016/j.snb.2020.129250.

[ref7] Wang F.; Gosling J. H.; Trindade G. F.; Rance G. A.; Makarovsky O.; Cottam N. D.; Kudrynskyi Z.; Balanov A. G.; Greenaway M. T.; Wildman R. D.; Hague R.; Tuck C.; Fromhold T. M.; Turyanska L. Inter-flake quantum transport of electrons and holes in inkjet-printed graphene devices. Adv. Funct. Mater. 2021, 31 (5), 2007478 10.1002/adfm.202007478.

[ref8] Austin J. S.; Cottam N. D.; Zhang C.; Wang F.; Gosling J. H.; Nelson-Dummet O.; James T. S. S.; Beton P. H.; Trindade G. F.; Zhou Y.; Tuck C. J.; Hague R.; Makarovsky O.; Turyanska L. Photosensitisation of inkjet printed graphene with stable all-inorganic perovskite nanocrystals. Nanoscale 2023, 15 (5), 2134–2142. 10.1039/D2NR06429D. 36644953

[ref9] Ávila J.; Momblona C.; Boix P. P.; Sessolo M.; Bolink H. J. Vapor-deposited perovskites: the route to high-performance solar cell production?. Joule 2017, 1 (3), 431–442. 10.1016/j.joule.2017.07.014.

[ref10] Kasparek C.; Blom P. W. M. Solution-processed multilayer polymer light-emitting diode without intermixing. Appl. Phys. Lett. 2017, 110 (2), 023302 10.1063/1.4973989.

[ref11] Sun J.; Bao B.; He M.; Zhou H.; Song Y. Recent Advances in Controlling the Depositing Morphologies of Inkjet Droplets. ACS Appl. Mater. Interfaces 2015, 7 (51), 28086–28099. 10.1021/acsami.5b07006. 26642390

[ref12] Singh M.; Haverinen H. M.; Dhagat P.; Jabbour G. E. Inkjet printing - process and its applications. Adv. Mater. 2010, 22 (6), 673–685. 10.1002/adma.200901141. 20217769

[ref13] Baeg K.-J. Polymer dielectrics and orthogonal solvent effects for high-performance inkjet-printed top-gated p-channel polymer field-effect transistors. ETRI J. 2011, 33 (6), 887–896. 10.4218/etrij.11.0111.0321.

[ref14] Zuniga C. A.; Barlow S.; Marder S. R. Approaches to solution-processed multilayer organic light-emitting diodes based on cross-linking. Chem. Mater. 2011, 23 (3), 658–681. 10.1021/cm102401k.

[ref15] Niu Y.-H.; Liu M. S.; Ka J.-W.; Jen A. K. Y. Thermally crosslinked hole-transporting layers for cascade hole-injection and effective electron-blocking/exciton-confinement in phosphorescent polymer light-emitting diodes. Appl. Phys. Lett. 2006, 88 (9), 093505 10.1063/1.2180882.

[ref16] Chu V. B.; Siopa D.; Debot A.; Adeleye D.; Sood M.; Lomuscio A.; Melchiorre M.; Guillot J.; Valle N.; El Adib B.; Rommelfangen J.; Dale P. J. Waste- and Cd-free inkjet-printed Zn(O,S) buffer for Cu(In,Ga)(S,Se)_2_ thin-film solar cells. ACS Appl. Mater. Interfaces 2021, 13 (11), 13009–13021. 10.1021/acsami.0c16860. 33689261

[ref17] Xie M.; Lu H.; Zhang L.; Wang J.; Luo Q.; Lin J.; Ba L.; Liu H.; Shen W.; Shi L.; Ma C. Fully solution-processed semi-transparent perovskite solar cells with ink-jet printed silver nanowires top electrode. Sol. RRL 2018, 2 (2), 1700184 10.1002/solr.201700184.

[ref18] Wei X.; Zhao L.; Wang J.; Zeng Y.; Li J. Characterization of nitride-based LED materials and devices using TOF-SIMS. Surf. Interface Anal. 2014, 46 (S1), 299–302. 10.1002/sia.5634.

[ref19] Mo H.-W.; Shirakura D.; Harada K.; Ishibashi K.; Shibamori T.; Miyamoto T.; Adachi C. Analyzing the degradation process of quantum-dot LEDs (QLEDs) by mass spectometry. SID Symp. Dig. Technol. Pap. 2022, 53 (1), 1–4. 10.1002/sdtp.15815.

[ref20] Trindade G. F.; Sul S.; Kim J.; Havelund R.; Eyres A.; Park S.; Shin Y.; Bae H. J.; Sung Y. M.; Matjacic L.; Jung Y.; Won J.; Jeon W. S.; Choi H.; Lee H. S.; Lee J.-C.; Kim J.-H.; Gilmore I. S. Direct identification of interfacial degradation in blue OLEDs using nanoscale chemical depth profiling. Nat. Commun. 2023, 14 (1), 8066 10.1038/s41467-023-43840-9. 38052834 PMC10698160

[ref21] Pious J. K.; Lai H.; Hu J.; Luo D.; Gilshtein E.; Siegrist S.; Kothandaraman R. K.; Lu Z. H.; Wolff C. M.; Tiwari A. N.; Fu F. In situ buried interface engineering towards printable Pb-Sn perovskite solar cells. ACS Appl. Mater. Interfaces 2024, 16 (30), 39399–39407. 10.1021/acsami.4c07083. 39031069

[ref22] Rivers G.; Austin J. S.; He Y.; Thompson A.; Gilani N.; Roberts N.; Zhao P.; Tuck C. J.; Hague R. J. M.; Wildman R. D.; Turyanska L. Stable large area drop-on-demand deposition of a conductive polymer ink for 3D-printed electronics, enabled by bio-renewable co-solvents. Addit. Manuf. 2023, 66, 103452 10.1016/j.addma.2023.103452.

[ref23] Brennan B.; Spencer S. J.; Belsey N. A.; Faris T.; Cronin H.; Silva S. R.; Sainsbury T.; Gilmore I. S.; Stoeva Z.; Pollard A. J. Structural, chemical and electrical characterisation of conductive graphene-polymer composite films. Appl. Surf. Sci. 2017, 403, 403–412. 10.1016/j.apsusc.2017.01.132.

[ref24] Morrison G. H.; Cheng K. L.; Grasserbauer M. General aspects of trace analytical methods-IV. Recommendations for Nomenclature, Standard Procedures and Reporting of Experimental Data for Surface Analysis Techniques. Pure Appl. Chem. 1979, 51 (11), 2243–2250. 10.1351/pac197951112243.

[ref25] Wang F.; Heaton C. E. D.; Cottam N. D.; Austin J. S.; Im J.; Fromhold M. T.; Wildman R. D.; Hague R. J. M.; Tuck C. J.; Makarovsky O.; Turyanska L. Inkjet printed multifunctional graphene sensors for flexible and wearable electronics. Adv. Electron. Mater. 2025, 2400689 10.1002/aelm.202400689.

[ref26] Jepiti P.; Yoon S.; Kim J. Electromigration reliability in Ag lines printed with nanoparticle inks: implications for printed electronics. ACS Appl. Nano Mater. 2022, 5 (2), 2569–2577. 10.1021/acsanm.1c04144.

[ref27] Jeong G.; Koo D.; Seo J.; Jung S.; Choi Y.; Lee J.; Park H. Suppressed interdiffusion and degradation in flexible and transparent metal electrode-based perovskite solar cells with a graphene interlayer. Nano Lett. 2020, 20 (5), 3718–3727. 10.1021/acs.nanolett.0c00663. 32223250

[ref28] Trindade G. F.; Wang F.; Im J.; He Y.; Balogh A.; Scurr D.; Gilmore I.; Tiddia M.; Saleh E.; Pervan D.; Turyanska L.; Tuck C. J.; Wildman R.; Hague R.; Roberts C. J. Residual polymer stabiliser causes anisotropic electrical conductivity during inkjet printing of metal nanoparticles. Commun. Mater. 2021, 2 (1), 47 10.1038/s43246-021-00151-0.

[ref29] Havelund R.; Seah M. P.; Tiddia M.; Gilmore I. S. SIMS of organic materials -interface location in argon gas cluster depth profiles using negative secondary ions. J. Am. Soc. Mass Spectrom. 2018, 29 (4), 774–785. 10.1007/s13361-018-1905-2. 29468500 PMC5889422

[ref30] Osaka M.; Mori D.; Benten H.; Ogawa H.; Ohkita H.; Ito S. Charge transport in intermixed regions of all-polymer solar cells studied by conductive atomic force microscopy. ACS Appl. Mater. Interfaces 2017, 9 (18), 15615–15622. 10.1021/acsami.7b00979. 28437063

[ref31] Ahn D. A.; Lee S.; Chung J.; Park Y.; Suh M. C. Impact of interface mixing on the performance of solution processed organic light emitting diodes - impedance and ultraviolet photoelectron spectroscopy study. ACS Appl. Mater. Interfaces 2017, 9 (27), 22748–22756. 10.1021/acsami.7b03557. 28632989

[ref32] Lee J. Y.; Ju B. K.; Cho K. H. Co-solvented solution filling and interfacial phenomena of sublimation transferred emitting layer for high-resolution OLED fabrication. APL Mater. 2021, 9 (10), 101115 10.1063/5.0058994.

[ref33] Yang J. N.; Chen T.; Ge J.; Wang J. J.; Yin Y. C.; Lan Y. F.; Ru X. C.; Ma Z. Y.; Zhang Q.; Yao H. B. High color purity and efficient green light-emitting diode using perovskite nanocrystals with the size overly exceeding Bohr exciton diameter. J. Am. Chem. Soc. 2021, 143 (47), 19928–19937. 10.1021/jacs.1c09948. 34766754

[ref34] Passarelli M. K.; Pirkl A.; Moellers R.; Grinfeld D.; Kollmer F.; Havelund R.; Newman C. F.; Marshall P. S.; Arlinghaus H.; Alexander M. R.; West A.; Horning S.; Niehuis E.; Makarov A.; Dollery C. T.; Gilmore I. S. The 3D OrbiSIMS-label-free metabolic imaging with subcellular lateral resolution and high mass-resolving power. Nat. Methods 2017, 14 (12), 1175–1183. 10.1038/nmeth.4504. 29131162

[ref35] ImageJ. https://imagej.nih.gov/ij/index.html (accessed Jun 29, 2022).

[ref36] Malmberg P.; Börner K.; Chen Y.; Friberg P.; Hagenhoff B.; Månsson J.-E.; Nygren H. Localization of lipids in the aortic wall with imaging TOF-SIMS. Biochimica et Biophysica Acta (BBA) - Molecular and Cell Biology of Lipids 2007, 1771 (2), 185–195. 10.1016/j.bbalip.2006.12.003. 17240191

[ref37] Isaacs M. A.; Davies-Jones J.; Davies P. R.; Guan S.; Lee R.; Morgan D. J.; Palgrave R. Advanced XPS characterization: XPS-based multi-technique analyses for comprehensive understanding of functional materials. Mater. Chem. Front. 2021, 5, 7931–7963. 10.1039/D1QM00969A.

