# Supplementary material for: Inkjet Printing of Heterostructures: Investigation and Strategies for Control of Interfaces
Source: ACS Appl Mater Interfaces. 2025 Mar 7;17(11):17230–7. doi: 10.1021/acsami.4c21170 (PMC11931478; doi:10.1021/acsami.4c21170)
Supplement: Supplementary file 1 — am4c21170_si_001.pdf [file am4c21170_si_001.pdf]

Supporting Information

Inkjet Printing of Heterostructures: Investigation and Strategies for Control of Interfaces

Jonathan S. Austin,[1] *Yundong Zhou,*[2] *Geoffrey Rivers* [1] *Negar Gilan,*[1] *Feiran Wang,*[1] *Christopher J. Tuck,*[1] *Ian S. Gilmore,*[2] *Richard J. M. Hague,*[1] *Gustavo F. Trindade*[2]* *and Lyudmila Turyanska*[1]*

[1]Centre for Additive Manufacturing, Faculty of Engineering, University of Nottingham, Jubilee Campus, Nottingham, NG8 1BB, UK

[2]National Physical Laboratory, Teddington, Middlesex, TW11 0LW, United Kingdom

*Corresponding Authors: gustavo.trindade@npl.co.uk;

Lyudmila.Turyanska@nottingham.ac.uk

SI1: Characterization of iGr/PEDOT:PSS heterostructures

ToF-SIMS spectra were recorded in negative polarity mode to map iGr/PEDOT:PSS (**Figure S1**). The $C_8H_7SO_3^-$ signal was used to map the PEDOT:PSS and F^- signal to map the graphene. The F^- signal was selected due to significantly smaller overlap with PEDOT:PSS signals, compared to C_6^- and C_2^- (**Figure S1**, bottom insets). Inorganic impurities, such as F, are likely introduced to graphene during the exfoliation process and are commonly used as marker signals in SIMS for carbon-based materials due to their ease of ionisation [S1]. ToF-SIMS depth profiles were measured with different fields of view (FOV) (with the same raster size of 300 × 300 µm) on the same sample, confirming that a change in the FOV from 200 × 200 µm to 32 × 32 µm had no significant effect on the results (**Figure S2**).

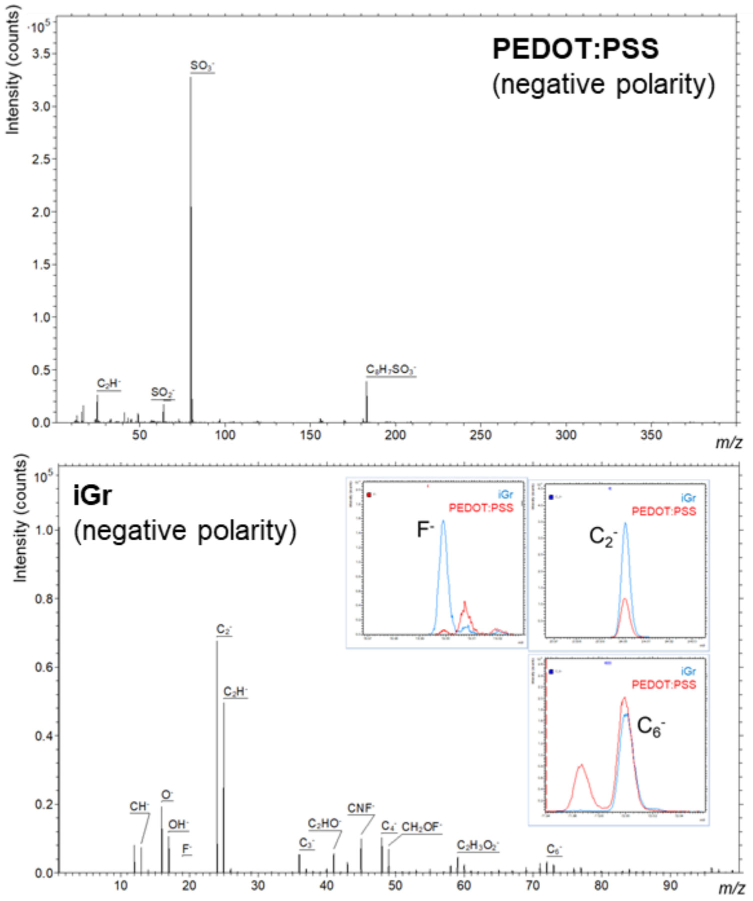

Figure S1. Representative ToF-SIMS spectra of PEDOT:PSS (top) and iGr (bottom) inks in negative polarity mode.

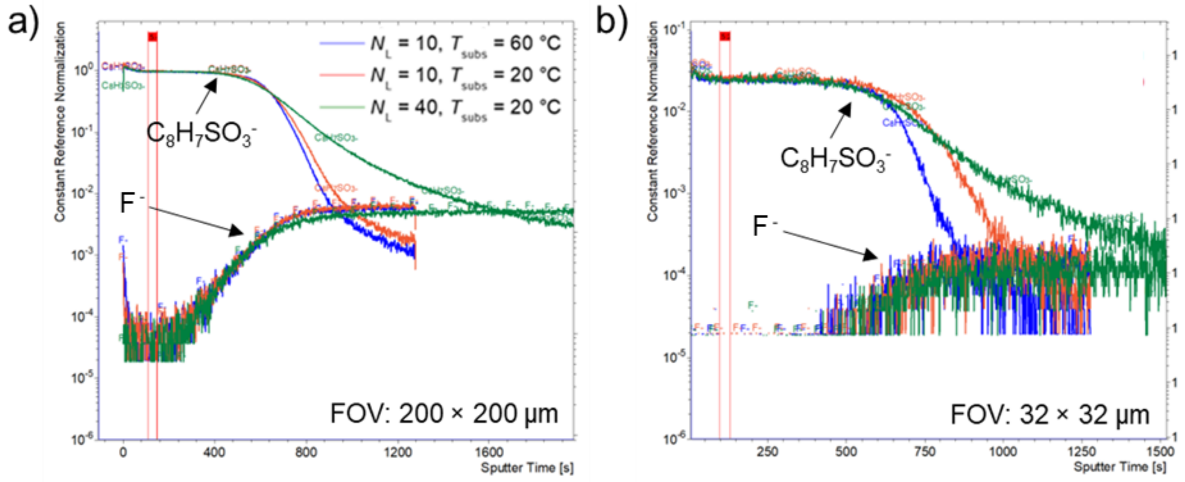

Figure S2. ToF-SIMS depth profiles of iGr/PEDOT:PSS heterostructures with iGr printed under different conditions **a)** measured with a 200 × 200 µm field of view and **b)** measured with a 32 × 32 µm field of view. ($C_8H_7O_3^-$) signal represents PEDOT:PSS and (F^-) represents iGr.

By reducing the polysorbate-80 concentration in the liquid PEDOT:PSS ink, we observed that intermixing between PEDOT:PSS and iGr was reduced, and thus a sharper PEDOT:PSS/iGr interface was achieved. A decrease from the typical 0.67 wt% (**Figure S3**, top) to 0.30 wt% (**Figure S3**, bottom) of surfactant content in the PEDOT:PSS ink was shown to reduce penetration of PEDOT:PSS into the iGr layer, from ΔX = 170 ± 40 nm to ΔX = 90 ± 20 nm while still providing sufficient surface tension adjustment to allow reliable printing of the PEDOT:PSS. For both inks, the polysorbate-80 surfactant was seen to accumulate at the PEDOT:PSS/iGr interface (**Figure S3**, pink lines), however, reducing the surfactant content to 0.30 wt% led to a narrowing of the oleic ion signal associated with the polysorbate-80, reducing its infiltration into the annealed iGr.

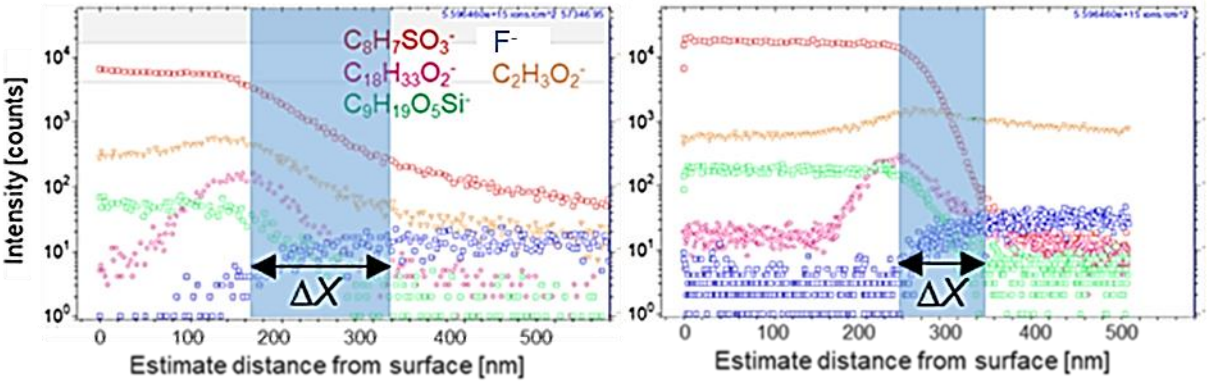

Figure S3. Depth profiles of iGr/PEDOT:PSS heterostructures (10 layers of each material printed on Kapton) using PEDOT:PSS inks with Tween-80 surfactant content of 0.67 wt% (left) and 0.30 wt% (right), measured via ToF-SIMS in negative polarity mode. $C_9H_{19}O_5Si^-$ (GOPS) and $C_8H_7SO_3^-$ correspond to components of PEDOT:PSS. ($C_{18}H_{33}O_2^-$) signal represents polysorbate-80 in PEDOT:PSS and (F^-) represents iGr.

FIB-SEM images of iGr (**Figure S4a**, top) revealed that porosity was present within the printed layers. The cross-sectional area of the pores, *A*, was estimated using ImageJ software (**Figure S4a**, middle and bottom). The estimates by areal pore fraction and the numerical pore fraction were comparable (**Figure S4b**), and the sheet resistance value were comparable (**Figure S4c**) for the samples produced with different printing parameters.

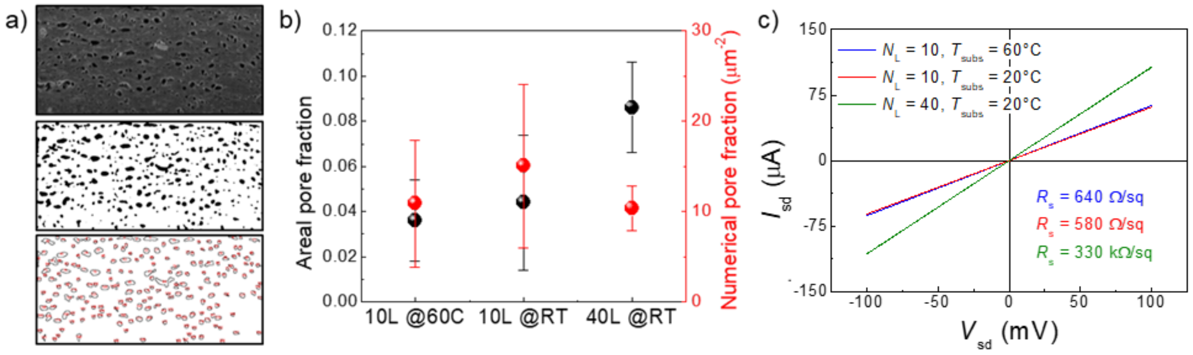

Figure S4. a) Exemplar FIB-SEM cross sectional image of iGr (top) followed by the thresholding process (middle) and the pore counting and measuring process (bottom) performed in ImageJ. **b)** Graph showing areal pore fraction (total area of pores divided by total area analysed) and numerical pore fraction (number of pores per unit area) of iGr printed under different conditions. Error bars are the standard deviation of four analysed FIB-SEM images. **c)** *I*(*V*) relationships of iGr printed under different conditions, with sheet resistance, R_s shown.

I(*V*) dependences were measured for 10 printed layers of iGr on Kapton (printed at room temperature) before (**Figure S5**, black) and after (**Figure S5**, red) the inkjet deposition of 10 printed layers of PEDOT:PSS. This revealed that the sheet resistance of the iGr is not significantly changed after PEDOT:PSS deposition, with < 10 % change in sheet resistance recorded across 3 repeat samples.

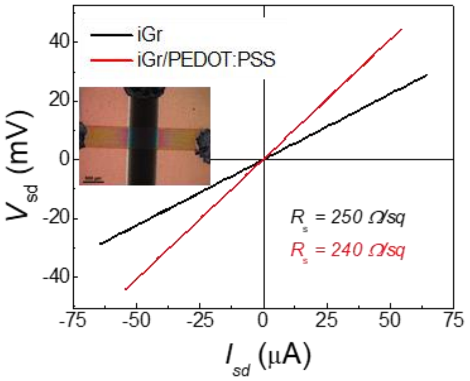

Figure S5. *I*(*V*) relationship of iGr (N_L = 10) before and after the deposition of PEDOT:PSS (N_L = 10) on top. Sample printed on Kapton substrate.

SI2: Characterization of iGr/CsPbr$_3$ heterostructures

ToF-SIMS was used in positive polarity mode to map $CsPbBr_3$/iGr heterostructures. In each printed ink a variety of different ions were recorded, as observed in representative ToF-SIMS spectra (**Figure S6**). We used the Cs_2Br^+ signal to map the $CsPbBr_3$ and Al^+ signal to map the graphene. The $C_3H_5^+$ signal represents residual organic solvents.

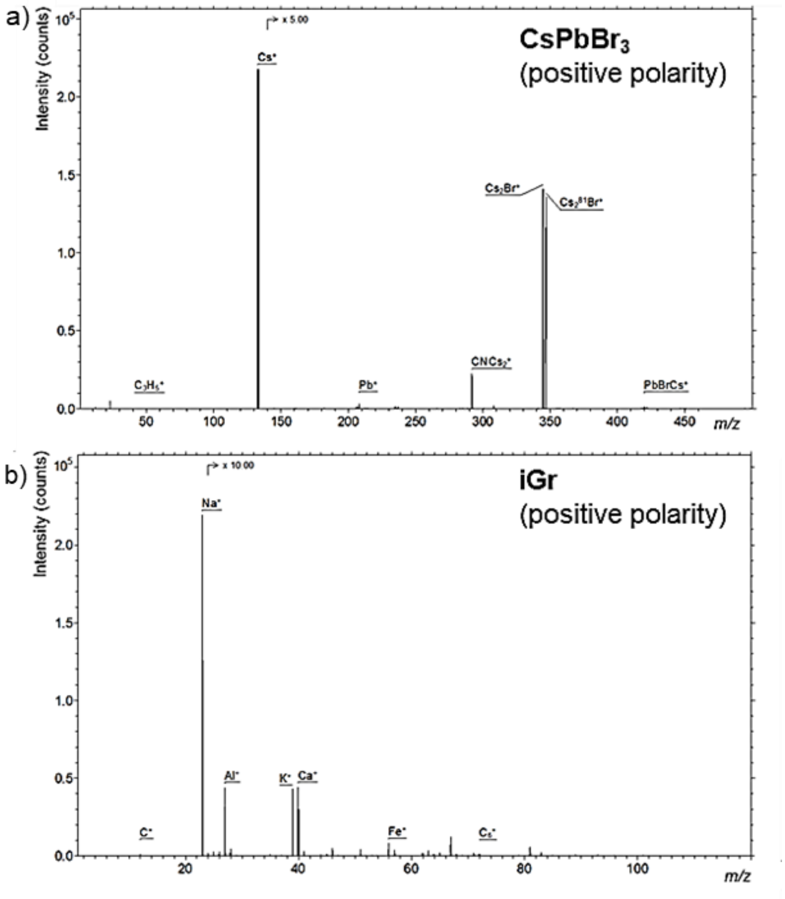

Figure S6. a) Representative ToF-SIMS spectra of $CsPbBr_3$ and **b)** iGr inks in positive polarity mode.

I(*V*) dependences were measured for N_L = 5 (**Figure S7a,** left) and N_L = 10 (**Figure S7a,** right) layers of iGr on Si/SiO_2 before and after the inkjet deposition of $CsPbBr_3$ perovskite with N_L = 5 (**Figure S7a,** left) and N_L = 10 (**Figure S7a,** right). This revealed that the sheet resistance of the iGr increase by ~ 60% following $CsPbBr_3$ NC deposition for the N_L = 5 layer sample. Interestingly, unlike iGr/$CsPbBr_3$ heterostructures, printing of $CsPbBr_3$-PVP into the iGr film

did not significantly affect the electrical properties of the iGr films (**Figure S8**), despite the insulating properties of the polymer-NC mixture.

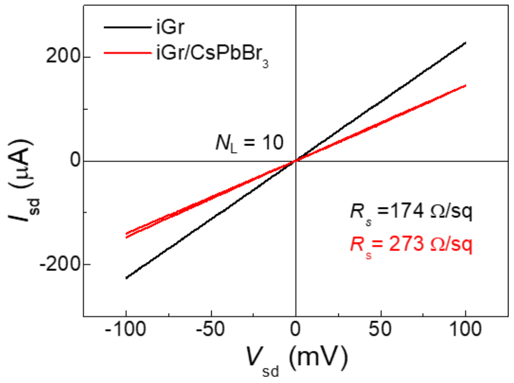

Figure S7. $I(V)$ relationships of iGr on Si/SiO_2, before and after inkjet deposition of $CsPbBr_3$ printed on top, with N_L = 10 layers of each material (iGr: T_{sub} = 60 °C, and T_{ann} = 250 °C for t = 2 hours; $CsPbBr_3$ T_{sub} = 60 °C, and T_{ann} = 60 °C for t = 30min)

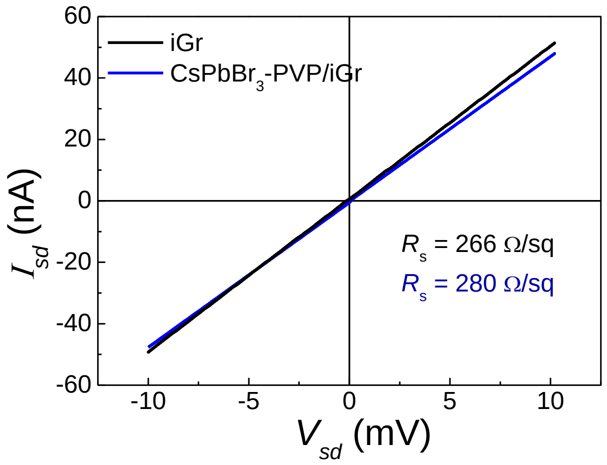

Figure S8. $I(V)$ relationships of iGr on Si/SiO_2, before and after inkjet deposition of $CsPbBr_3$-PVP printed on top, for N_L = 10 layers of each material (iGr: T_{sub} = 60 °C, and T_{ann} = 250 °C for t = 2 hours)

SI3: Characterization of iGr/PEDOT:PSS/CsPbr$_3$ Heterostructures

100-layer $CsPbBr_3$ was printed on top of 10 layers of PEDOT:PSS and iGr. EDX maps (**Figure S9**) were taken over the region shown in the large FIB-SEM image shown in **Figure 5b**. EDX mapping revealed the formation of a rough CuO layer on top of the Cu. Above this, the C signal indicates the iGr layer, the S signal indicates the PEDOT:PSS layer, and the Br and Pb signals indicate the $CsPbBr_3$ layer. These low resolution EDX maps suggest some overlap between the $CsPbBr_3$ and PEDOT:PSS layers, which may imply intermixing between them, but it is unclear if any intermixing occurs between the PEDOT:PSS and iGr layers from FIB-SEM and EDX alone due to the resolution, and inability to clearly dissociate the elemental chemistry.

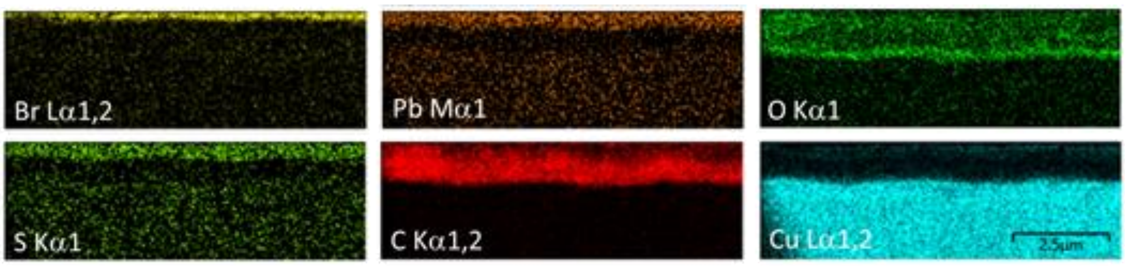

Figure S9. EDX maps of iGr/$CsPbBr_3$ heterostructure (100-layer $CsPbBr_3$, 10 layers of PEDOT:PSS and iGr).

The iGr layer within the iGr/PEDOT:PSS/$CsPbBr_3$ heterostructure (N_L = 10 layers, T_{sub} = 60 °C) displayed comparable pore size distribution (**Figure S10**, right) and average pore size A_{mean} = 2000 ± 300 nm^2 to the iGr layer from the iGr/PEDOT:PSS heterostructure printed using the same parameters (**Figure S10**, left) with A_{mean} = 2300 ± 200 nm^2.

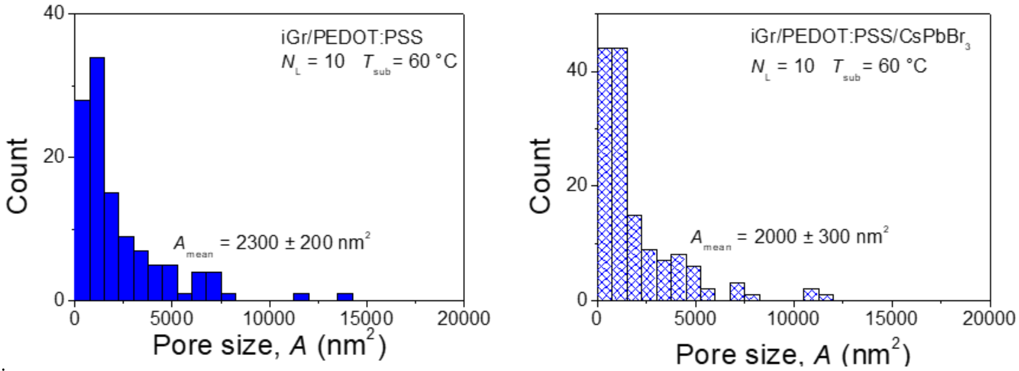

Figure S10. Histograms of the pore sizes within the iGr layers (N_L = 10 layers, T_{sub} = 60 °C) in iGr/PEDOT:PSS heterostructure (left) and iGr/PEDOT:PSS/$CsPbBr_3$ heterostructure (right).

Contact angle measurements of iGr ink revealed a contact angle of $\theta = 21°$ on Cu and $\theta = 26°$ on Si/SiO_2 substrates (**Figure S11**). This. The smaller contact angle on Cu, leads to greater ink spreading and larger print area. Hence, we observe thinner layers for the same n_L on Cu compared to Si/SiO_2.

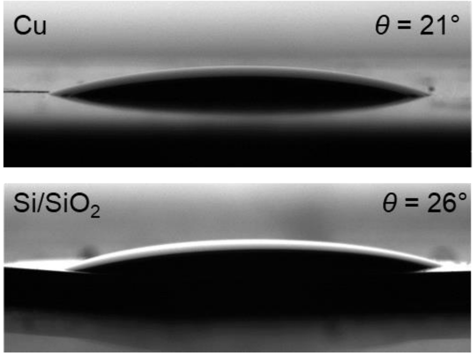

Figure S11. Optical images from drop-shape analyser of iGr ink droplet on Cu substrate (top) and Si/SiO_2 substrate (bottom)

ToF-SIMS was used in negative polarity for iGr/PEDOT:PSS/$CsPbBr_3$ heterostructures (**Figure S1** and **Figure S12**), with $PbBr^-$ signal, $C_8H_7SO_3^-$signal and F^- signal to map the $CsPbBr_3$, PEDOT:PSS and iGr, respectively. No significant change of iGr sheet resistance was observed following the deposition of PEDOT:PSS and $CsPbBr_3$ NCs (**Figure S13**).

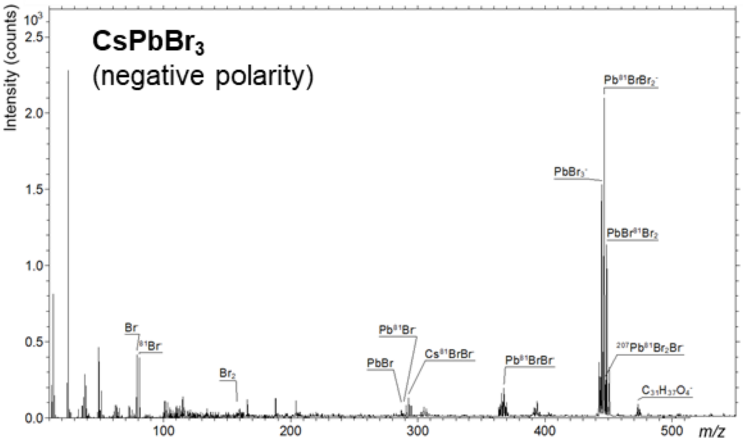

Figure S12. Representative ToF-SIMS spectra of $CsPbBr_3$ ink in negative polarity mode.

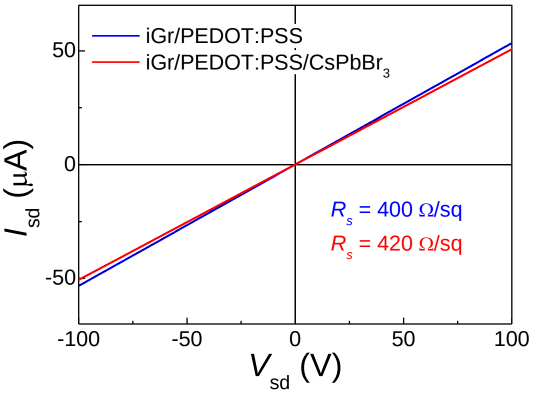

Figure S13. I(V) relationships of iGr on Si/SiO_2 (T_{sub} = 60 °C, and T_{ann} = 250 °C for t = 2 hours) in iGr/PEDOT:PSS heterostructure (PEDOT:PSS: N_L = 10 layers, T_{sub} = 45 °C, and T_{ann} = 150 °C for t = 30 min) (blue line) and in iGr/PEDOT:PSS/$CsPbBr_3$ heterostructure ($CsPbBr_3$: NL = 100 layers, T_{sub} = 60 °C, and T_{ann} = 60 °C for t = 30 mins).

REFERENCES

[S1] B. Brennan, S.J. Spencer, N.A. Belsey, T. Faris, H. Cronin, S.R. Silva, T. Sainsbury, I.S. Gilmore, Z. Stoeva, A.J. Pollard, Structural, chemical and electrical characterisation of conductive graphene-polymer composite films, Appl. Surf. Sci. 403 (2017). https://doi.org/10.1016/j.apsusc.2017.01.132
